# Supplementary material for: Multi-Level Optimization and Strategies in Microbial Biotransformation of Nature Products
Source: Molecules. 2023 Mar 14;28(6):2619. doi: 10.3390/molecules28062619 (PMC10051863; doi:10.3390/molecules28062619)

Supplementary Materials

Multi-level Optimization and Strategies in Microbial Biotransformation of Nature Products

FigureS1. Structural formula and synthesis route of all compounds in Table 1.

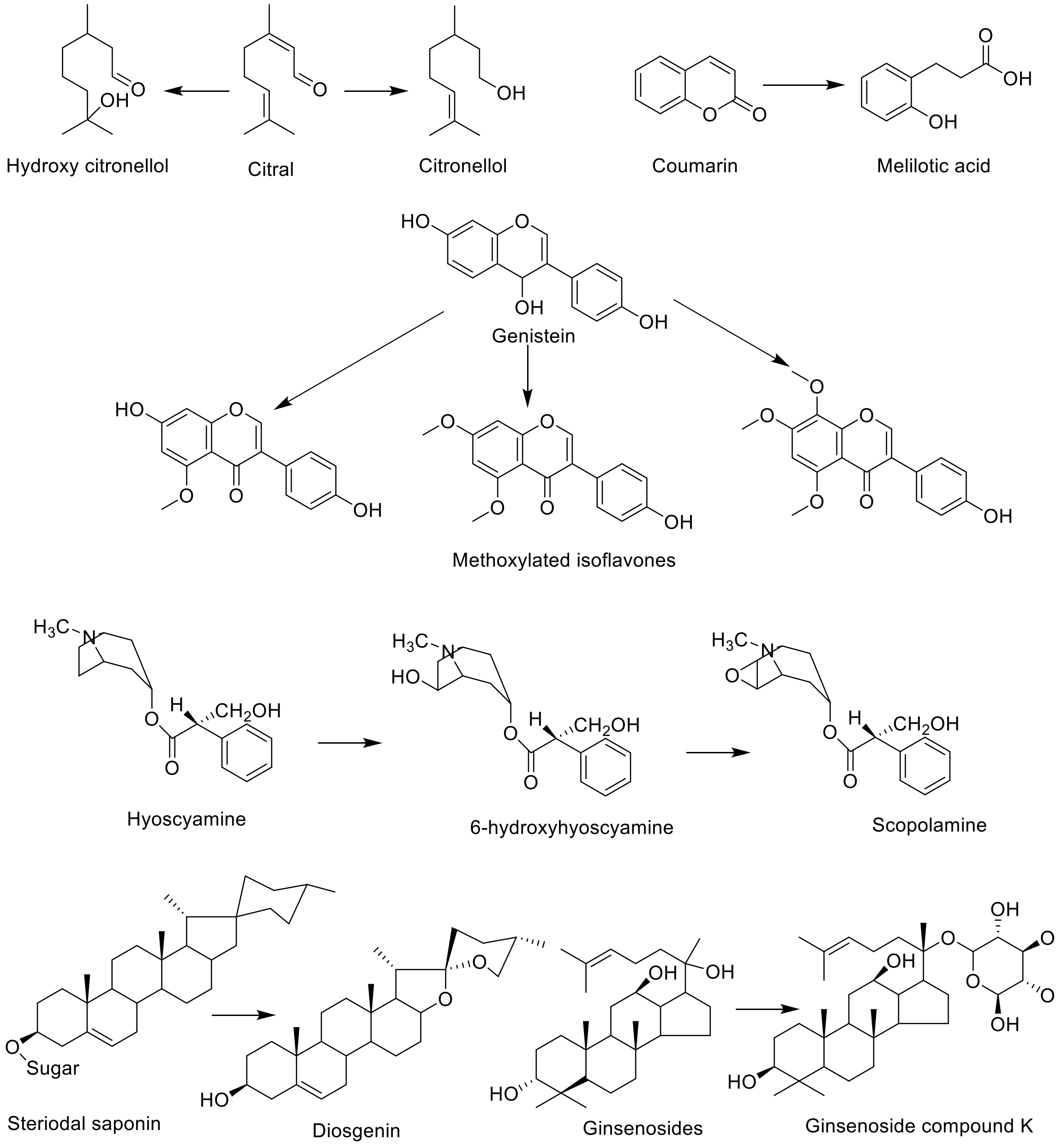

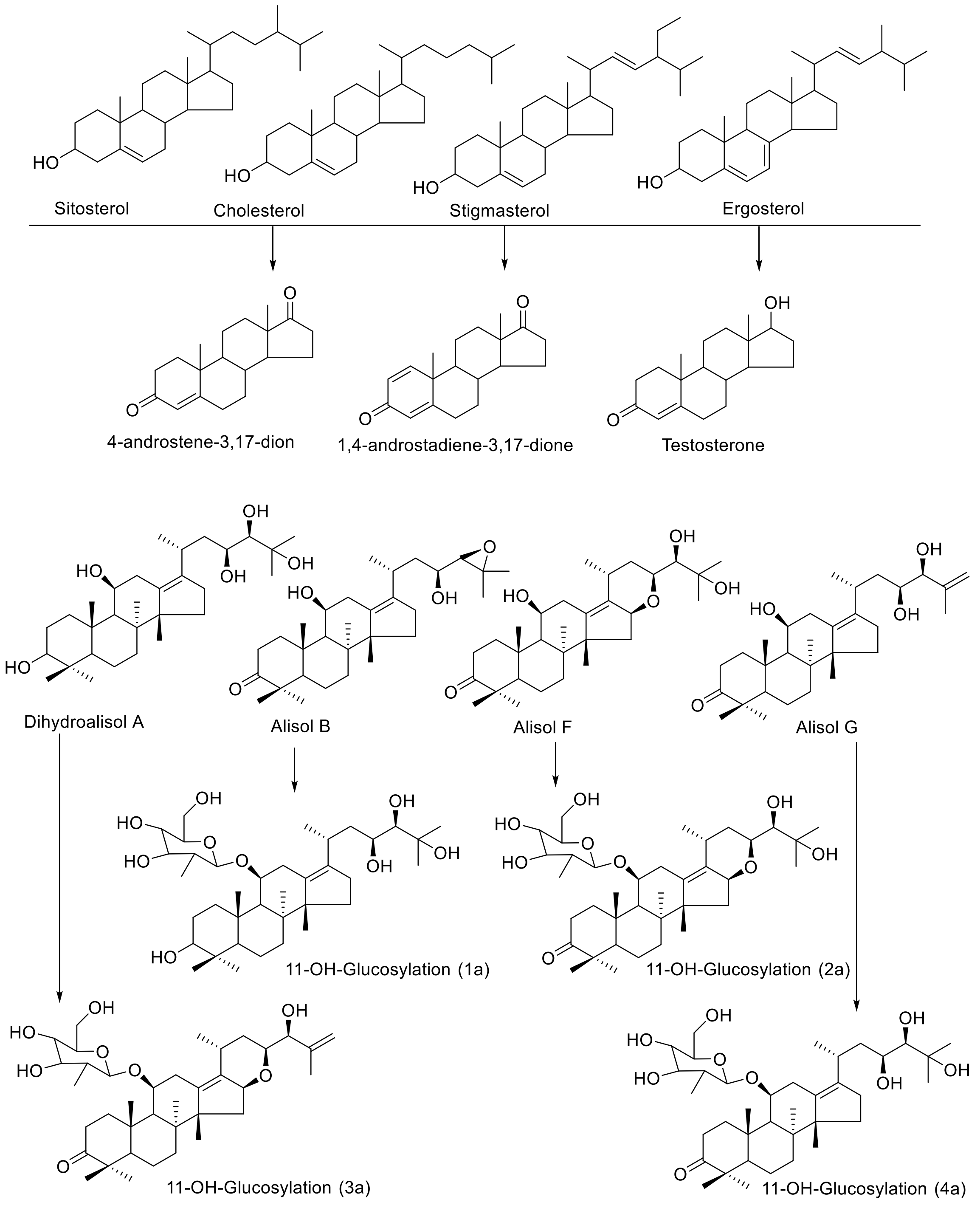
Sitosterol
Cholesterol
Stigmasterol
Ergosterol
4-androstene-3,17-dion
1,4-androstadiene-3,17-dione
Testosterone
Dihydroalisol A
Alisol B
Alisol F
Alisol G
11-OH-Glucosylation (1a)
11-OH-Glucosylation (2a)
11-OH-Glucosylation (3a)
11-OH-Glucosylation (4a)

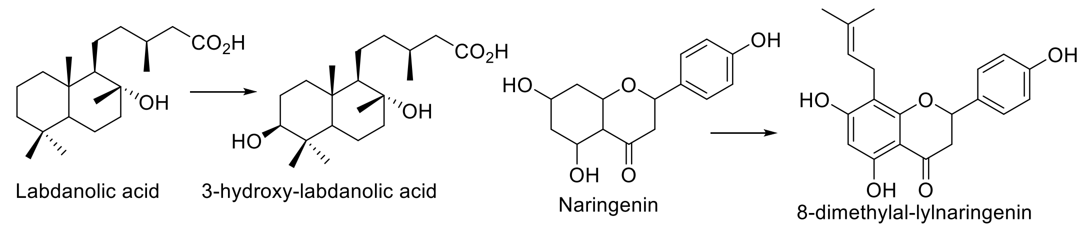

Labdanolic acid — 3-hydroxy-labdanolic acid — Naringenin — 8-dimethylal-lylnaringenin

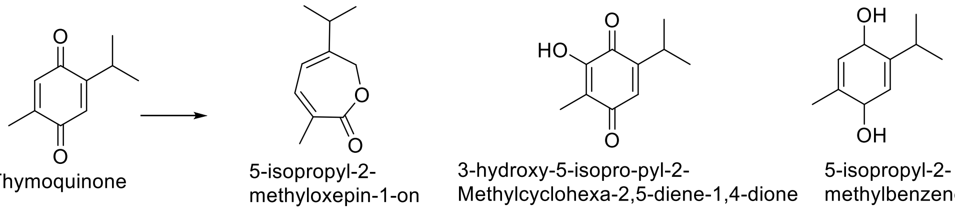

Thymoquinone — 5-isopropyl-2-methyloxepin-1-on — 3-hydroxy-5-isopro-pyl-2-Methylcyclohexa-2,5-diene-1,4-dione — 5-isopropyl-2-methylbenzene-1,4-diol

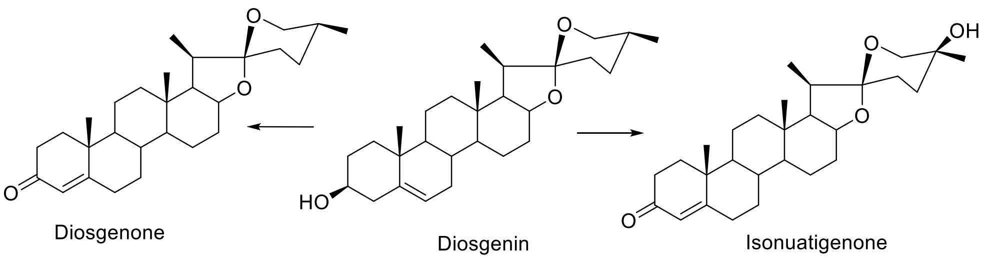

Diosgenone — Diosgenin — Isonuatigenone

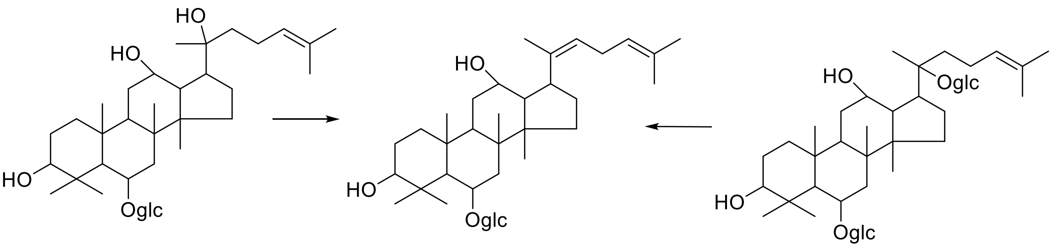

Ginsenoside Rh1 — Gingsenoside Rh4 — Ginsenoside Re

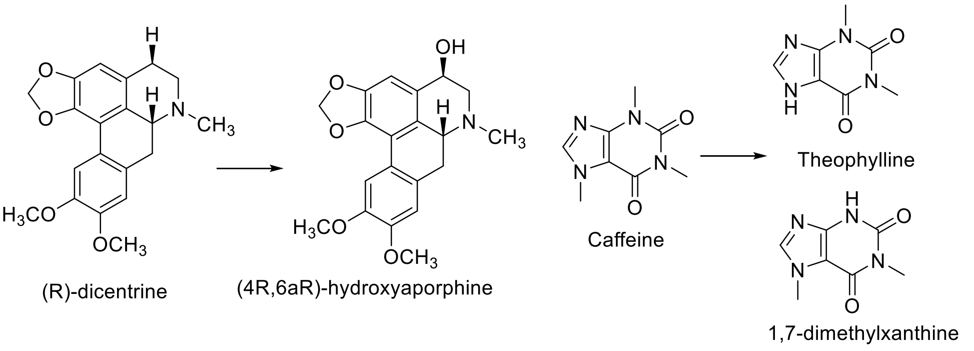

(R)-dicentrine — (4R,6aR)-hydroxyaporphine — Caffeine — Theophylline — 1,7-dimethylxanthine

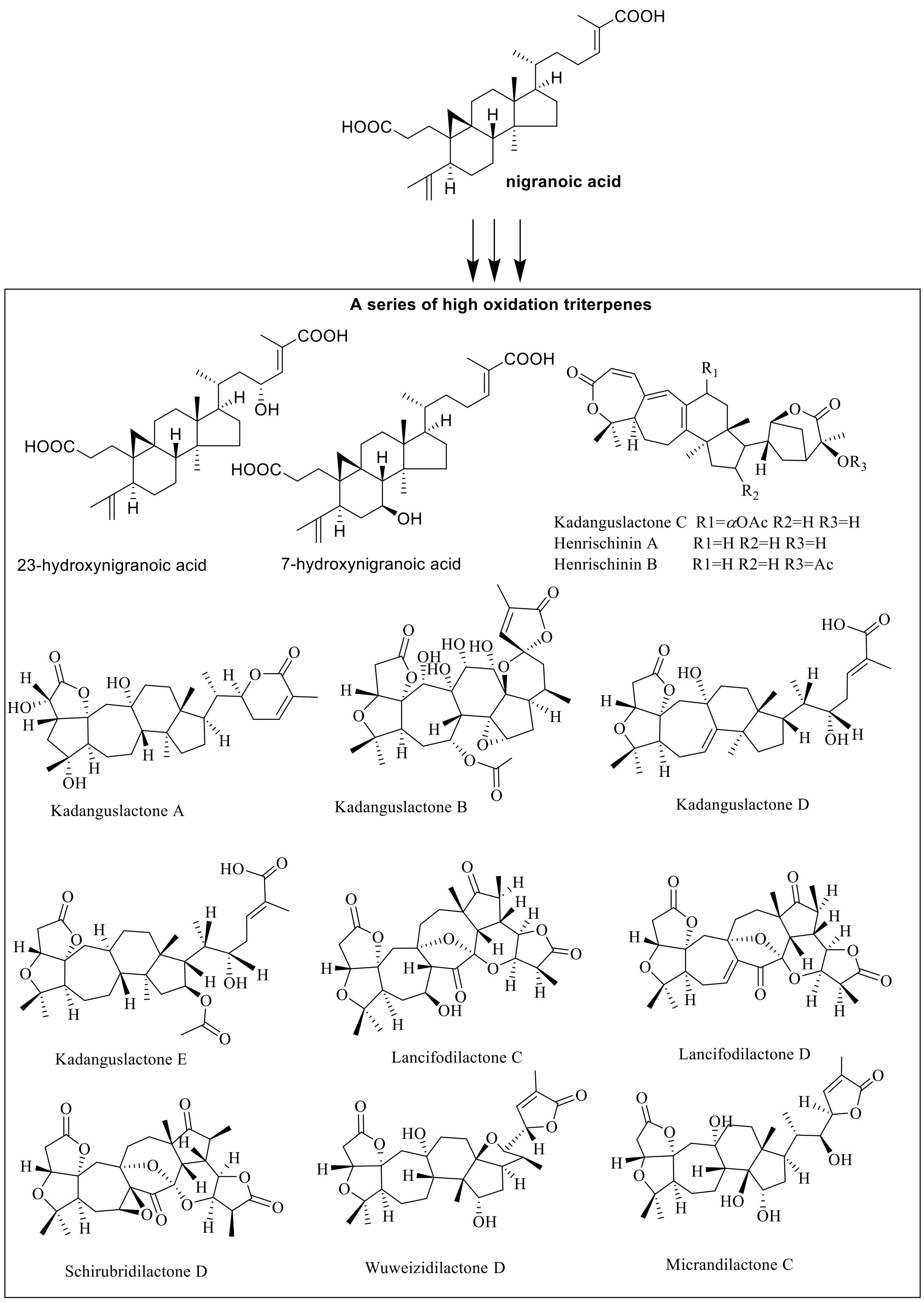

FigureS2. Structural formula and synthesis route of all compounds in Table 2.

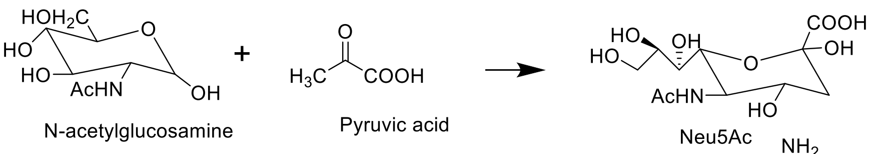
N-acetylglucosamine
Pyruvic acid
Neu5Ac

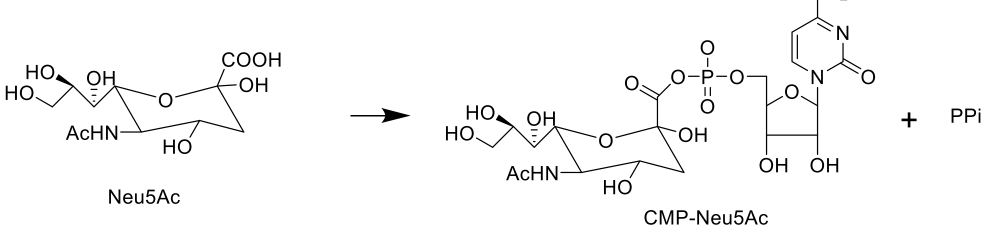
Neu5Ac
CMP-Neu5Ac
PPi

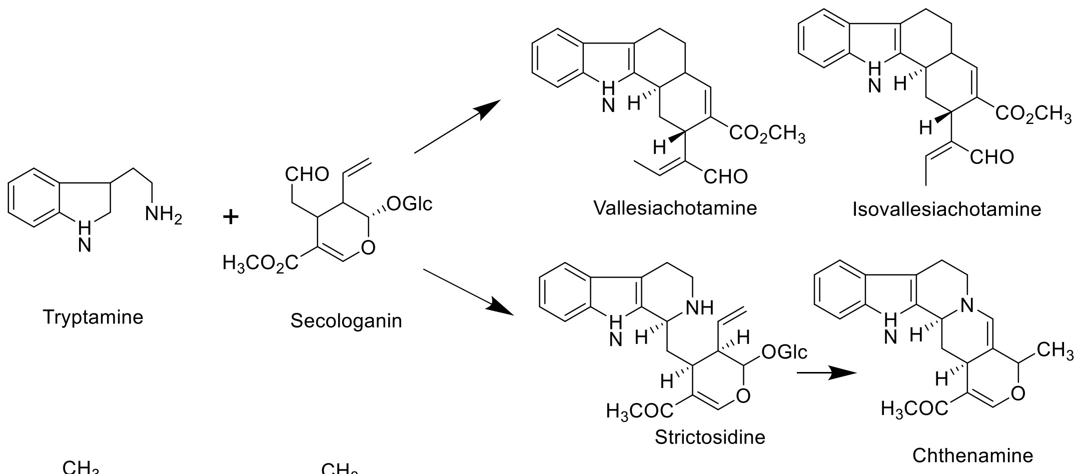
Tryptamine
Secologanin
Vallesiachotamine
Isovallesiachotamine
Strictosidine
Chthenamine

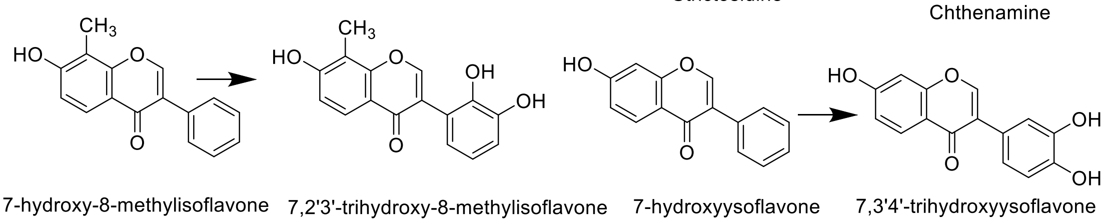
7-hydroxy-8-methylisoflavone
7,2'3'-trihydroxy-8-methylisoflavone
7-hydroxyysoflavone
7,3'4'-trihydroxyysoflavone

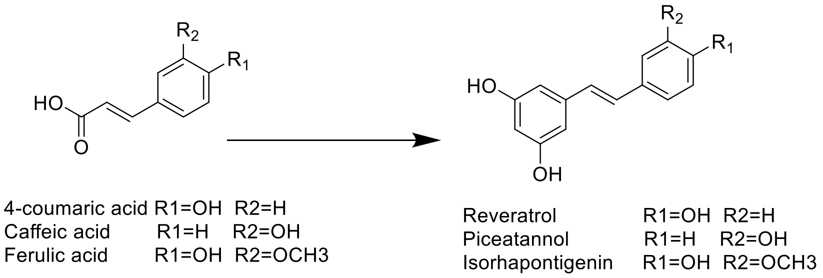
4-coumaric acid R1=OH R2=H
Caffeic acid R1=H R2=OH
Ferulic acid R1=OH R2=OCH3
Reveratrol R1=OH R2=H
Piceatannol R1=H R2=OH
Isorhapontigenin R1=OH R2=OCH3

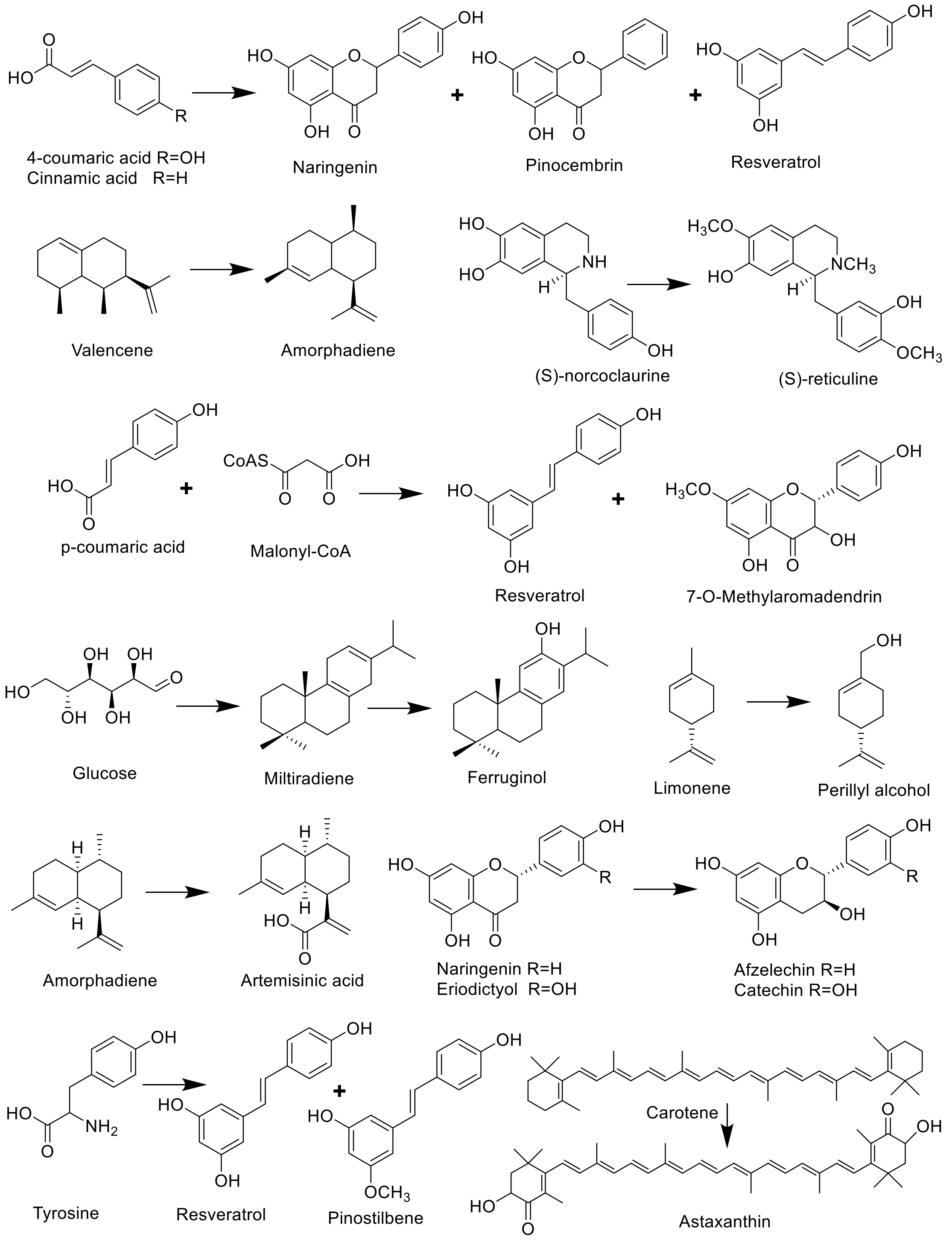
4-coumaric acid R=OH
Cinnamic acid R=H
Naringenin
Pinocembrin
Resveratrol
Valencene
Amorphadiene
(S)-norcoclaurine
(S)-reticuline
p-coumaric acid
Malonyl-CoA
Resveratrol
7-O-Methylaromadendrin
Glucose
Miltiradiene
Ferruginol
Limonene
Perillyl alcohol
Amorphadiene
Artemisinic acid
Naringenin R=H
Eriodictyol R=OH
Afzelechin R=H
Catechin R=OH
Tyrosine
Resveratrol
Pinostilbene
Carotene
Astaxanthin

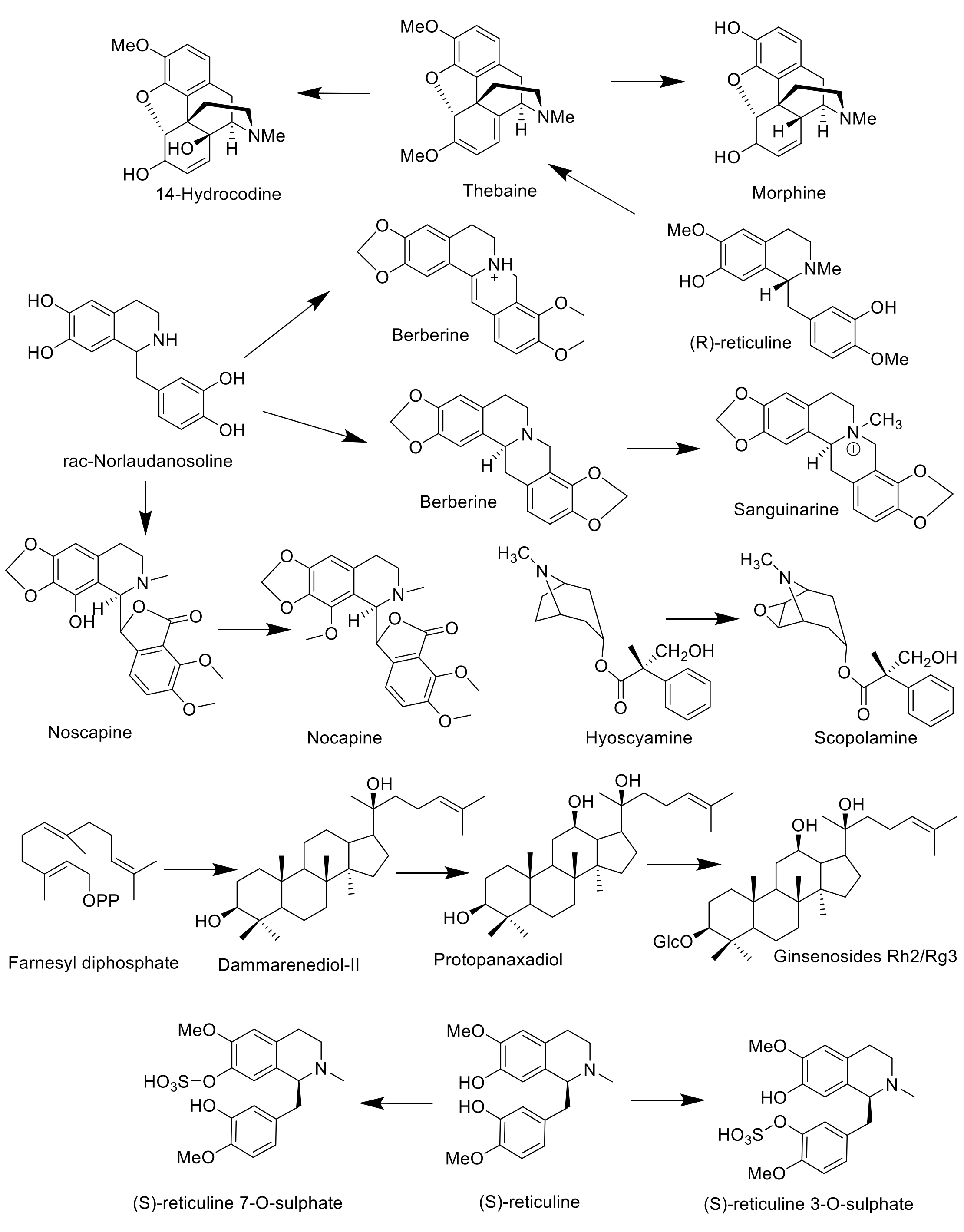
14-Hydrocodine
Thebaine
Morphine
Berberine
(R)-reticuline
rac-Norlaudanosoline
Berberine
Sanguinarine
Noscapine
Nocapine
Hyoscyamine
Scopolamine
Farnesyl diphosphate
Dammarenediol-II
Protopanaxadiol
Ginsenosides Rh2/Rg3
(S)-reticuline 7-O-sulphate
(S)-reticuline
(S)-reticuline 3-O-sulphate

FigureS3. Structural formula and synthesis route of all compounds in Table 3.

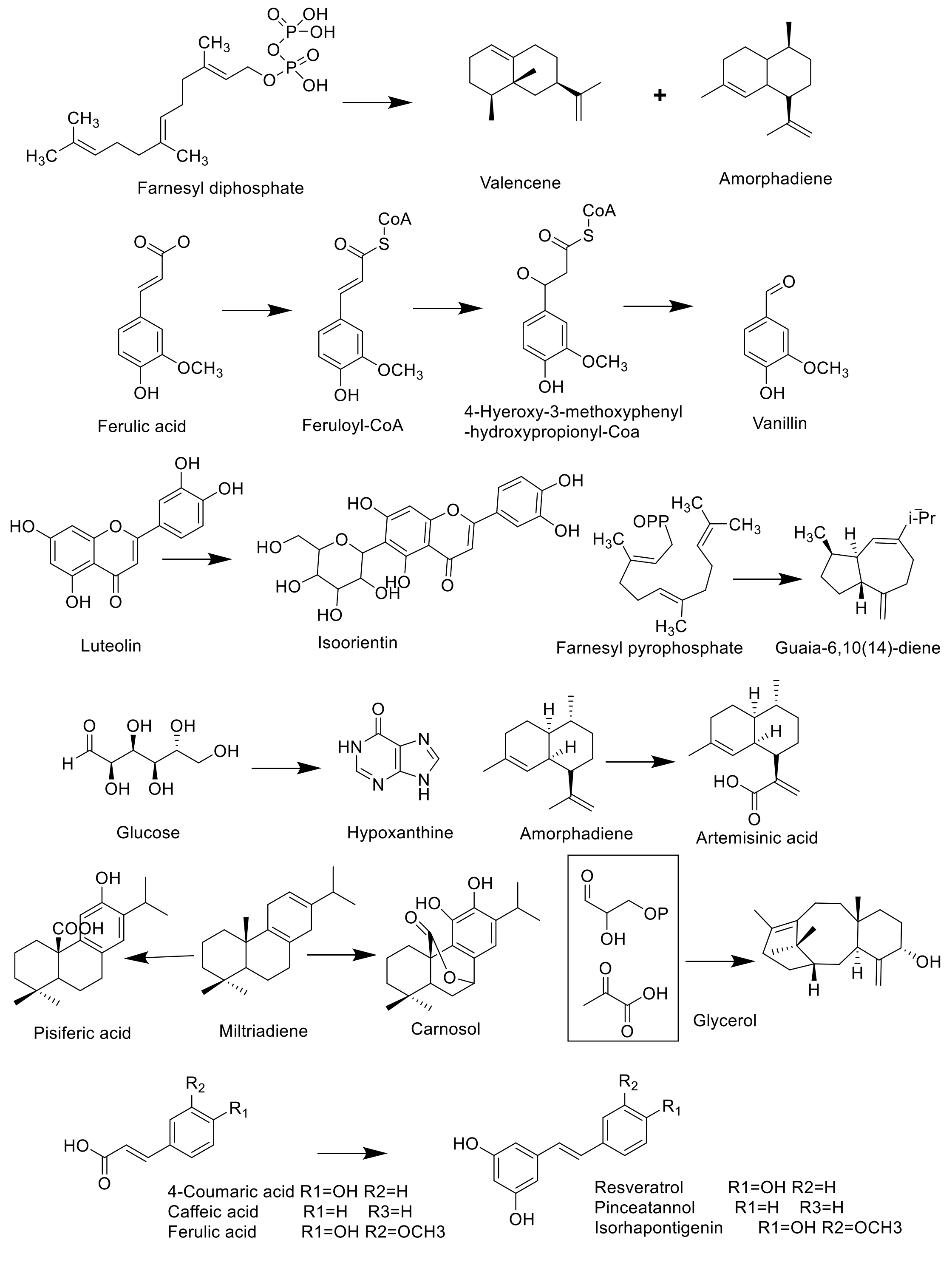

Supplement: Supplementary file 1 [file molecules-28-02619-s001.zip › molecules-2250869-supplementary.pdf]
